# Biliary Leak after Pediatric Liver Transplantation Treated by Percutaneous Transhepatic Biliary Drainage—A Case Series

**DOI:** 10.3390/tomography9050153

**Published:** 2023-10-19

**Authors:** Michael Doppler, Christin Fürnstahl, Simone Hammer, Michael Melter, Niklas Verloh, Hans Jürgen Schlitt, Wibke Uller

**Affiliations:** 1 Department of Diagnostic and Interventional Radiology, Medical Center—University of Freiburg, Faculty of Medicine, University of Freiburg, 79106 Freiburg, Germany; 2 Department of Radiology, University of Regensburg, University Medical Center Regensburg, 93053 Regensburg, Germany; 3 University Children’s Hospital Regensburg, University of Regensburg, University Medical Center Regensburg, 93053 Regensburg, Germany; 4 Department of Surgery, University of Regensburg, University Medical Center Regensburg, 93053 Regensburg, Germany

**Keywords:** PTBD, pediatric liver transplantation, biliary leak, pLTx, percutaneous transhepatic biliary drainage

## Abstract

Background: Biliary leaks are a severe complication after pediatric liver transplantation (pLT), and successful management is challenging. Objectives: The aim of this case series was to assess the outcome of percutaneous transhepatic biliary drainage (PTBD) in children with bile leaks following pLT. The necessity of additional percutaneous bilioma drainage and laboratory changes during therapy and follow-up was documented. Material and Methods: All children who underwent PTBD for biliary leak following pLT were included in this consecutive retrospective single-center study and analyzed regarding site of leak, management of additional bilioma, treatment response, and patient and transplant survival. The courses of inflammation, cholestasis parameters, and liver enzymes were retrospectively reviewed. Results: Ten children underwent PTBD treatment for biliary leak after pLT. Seven patients presented with leakage at the hepaticojejunostomy, two with leakage at the choledocho-choledochostomy and one with a bile leak because of an overlooked segmental bile duct. In terms of the mean, the PTBD treatment started 40.3 ± 31.7 days after pLT. The mean duration of PTBD treatment was 109.7 ± 103.6 days. Additional percutaneous bilioma drainage was required in eight cases. Bile leak treatment was successful in all cases, and no complications occurred. The patient and transplant survival rate was 100%. CRP serum level, leukocyte count, gamma-glutamyl transferase (GGT), and total and direct bilirubin level decreased significantly during treatment with a very strong effect size. Additionally, the gamma-glutamyl transferase level showed a statistically significant reduction during follow-up. Conclusions: PTBD is a very successful strategy for bile leak therapy after pLT.

## 1. Introduction

Liver transplantation is the only long-term option for treating end-stage liver disease in childhood. Reasons for end-stage liver disease in childhood may be cholestatic liver diseases, metabolic liver diseases with and without liver cirrhosis, infectious and non-infectious hepatitis, malignant and benign liver tumors, liver failure, Budd–Chiari syndrome, or cryptogenic liver cirrhosis.

Approximately 100 pediatric liver transplantations (pLTxs) are carried out in Germany every year. In 2020, 140 liver transplantations (pLTs) were performed in pediatric patients in Germany. The long-term survival rate (more than 5 years) is reported at 90% [1,2]. Approximately two-thirds of pediatric liver transplantations are composed of deceased-donor liver transplantations (DDLTs), and one-third are living-donor liver transplantations (LDLTs) [3]. In total, 60% of pLTs are full-size liver transplantations (FSLTs), 28.5% are split-liver transplantations (SLTs), and 11.5% are living-related liver transplantations (LRLTs) [4].

Complications after liver transplantation in childhood may occur acutely and in the long term. Frequently occurring complications are vascular complications or bowel perforation and bleeding events [5]. 

Numerous risk factors influence the occurrence of complications [6]. Thus, the morbidity rate after pediatric liver transplantation is also related to the type of transplantation [6]. Overall, complications occur much more frequently within the first 30 days after pLTxs after partial liver transplants than after whole-organ transplants [6].

Biliary complications after pediatric liver transplantation are also common events and occur in 10–35% of cases [6,7,8,9,10]. Biliary complications manifest as stenoses (up to 11% after pLT), insufficiencies (2–15% after pLT), biliomas (2.4% after pLT), detached bile ducts, gallstones, sludge, cast, or concrements [8,11,12]. Biliary complications after liver transplantation in childhood may include abdominal pain, fever, cholangitis, sepsis, or elevated cholestasis parameters [13]. However, patients may remain asymptomatic for a long time [13].

Biliary stenoses are divided into anastomotic stenoses and non-anastomotic biliary stenoses [13]. While anastomotic stenoses can develop only at biliodigestive anastomoses, non-anastomotic stenoses affect both intrahepatic and extrahepatic bile ducts [13]. The anastomotic stenoses occur in 10–35% of cases after pediatric liver transplantation and may be due to retraction or scarring, inadequate blood supply to the common/choledochal duct, or size inconsistency of the recipient and donor organs [13,14]. The non-anastomotic stenoses may be caused, first, by stenosis or thrombosis/occlusion of the hepatic artery, and second, there are non-anastomotic stenoses in unobstructed or non-stenotic hepatic arteries (ITBLs: Ischemic-Type Biliary Lesions), which may occur as a consequence of microangiopathic or immunogenic damage [13,15,16]. The occurrence of ITBLs is reported with a frequency of 1–19% after liver transplantation [15]. 

Biliary leaks occur in 2–15% of cases after liver transplantation in childhood, typically in the first three months after liver transplantation [8,17,18,19]. The leakage may be located at the biliodigestive anastomosis (Roux-en-Y hepaticojejunostomy or choledocho-choledochostomy), the liver cut surface of partial living-related grafts, and at the intrahepatic bile ducts. Small leaks may be managed by non-invasive treatment and merely leaving an intraoperatively placed drain in situ. However, more severe leaks are associated with higher morbidity rates, including cholangitis, abscess, inflammation, as well as graft loss, and require further therapy [14]. 

In general, endoscopic retrograde cholangiopancreatography (ERCP), with the option of papillotomy, is the method of choice for interventional treatment of the bile ducts.

Re-surgery is also possible in cases of biliary leakage and stenosis, e.g., with re-installation of the biliodigestive anastomosis [20].

Since hepaticojejunostomy with Roux-en-Y reconstruction is the most common type of biliary anastomosis in pediatric patients, endoscopic retrograde cholangiopancreatography (ERCP) is complicated for the treatment of biliary leaks and not possible in the early phase after liver transplantation. In this situation, percutaneous transhepatic treatment renders an alternative technique to endoscopic management and surgical revisions [21]. 

The aim of this study was to evaluate the long-term prognosis of liver transplantation in children with special reference to bile leaks and their treatment with PTBD. Study endpoints were closure of the leak and duration of PTBD placement, patient, and transplant survival. The course of inflammatory, cholestasis serum levels, and liver enzymes were analyzed as parameters for therapy monitoring.

## 2. Material and Methods

### 2.1. Study Design

This single-center, consecutive retrospective case series was conducted according to the principles expressed in the Declaration of Helsinki. Institutional review board approval was obtained. The requirement for informed consent was waived for this retrospective study. Inclusion criteria were PTBD procedures for managing biliary leaks following liver transplantation in children under 18 years. 

### 2.2. Procedure Technique

If biliary leak was clinically suspected but MRI did not reveal biliary leak, PTC was performed to confirm or rule out biliary leak. If biliary leak was confirmed by PTC, PTBD placement followed. 

Experienced interventional radiologists performed the procedures. Informed consent for the procedure was obtained. PTBD implantations were performed using a sterile technique in a standardized manner under general anesthesia. After the application of intravenous antibiotic prophylaxis, a Chiba needle (22G, Cook Medical, Bjaeverskov, Denmark) was advanced percutaneously into a peripheral bile duct under real-time ultrasound guidance. The biliodigestive anastomosis was passed with angled catheters and guide wires. Finally, a 6 F-internal–external drainage was inserted (Cook Medical, Bjaeverskov, Denmark). In the case of the unconnected central bile duct leaking to the jejunum, an external drain was placed until surgical repair. 

After two weeks, the internal–external drains were replaced with softer, Münchner drains (Peter Pflugbeil GmbH, Zorneding, Germany). Cholangiograms were carried out every 6 to 8 weeks, and the diameter of the drainages was gradually increased by 2 French (F) to achieve the final drainage size adapted to the children’s bodyweight. The side holes of the drainages were placed above and below the leakage to ensure optimal bile drainage. From 24 to 72 h after drainage placement or exchange, drains were closed to allow only internal drainage. All drains were rinsed with 5–10 mL sodium chloride once or twice daily to ensure optimal bile flow [22]. 

### 2.3. Definitions and Data

End of drainage therapy was defined as the absence of contrast leakage and stenosis (in case of additional stenosis) from the biliary system during cholangiography. Technical success was defined as successful PTBD placement, the complete resolution of biliary leakage proven at cholangiogram, and no recurrence of leakage during follow-up. 

Data were collected from electronic patient charts and a picture-achieving computer system (PACS). The analysis included clinical demographics, procedural data, and changes in laboratory parameters 1–7 days before and after PTBD treatment and during follow-up (glutamate oxaloacetate transaminase (GOT), glutamate pyruvate transaminase (GPT), glutamate dehydrogenase (GLDH), gamma-glutamyl transferase (GGT), alkaline phosphatase (ALP), cholinesterase (CHE), total, direct, and indirect bilirubin, C-reactive protein (CRP), leukocyte count, albumin, Quick, International Normalized Ratio (INR)). Follow-up was performed every 3 months after patient discharge in the first postprocedural year and thereafter annually. 

### 2.4. Statistical Analysis

Numerical variables were summarized using descriptive statistics. Laboratory parameters before, during, and after PTBD therapy were compared using the Wilcoxon sign rank test. A *p* < 0.05 was considered statistically significant. 

The following formula was used to calculate the effect size (r): r = |z:√n|. To assess the magnitude of the effect, Cohen’s classification from 1988 was used [23]. An |r| of 0.1 to <0.3 corresponds to a small effect, an |r| of 0.3 to <0.5 to a medium effect, and an |r| of 0.5 or greater to a strong effect. Analysis was performed using SPSS (Version 25.0). 

## 3. Results

### 3.1. Study Cohort

**Scheme 1:**
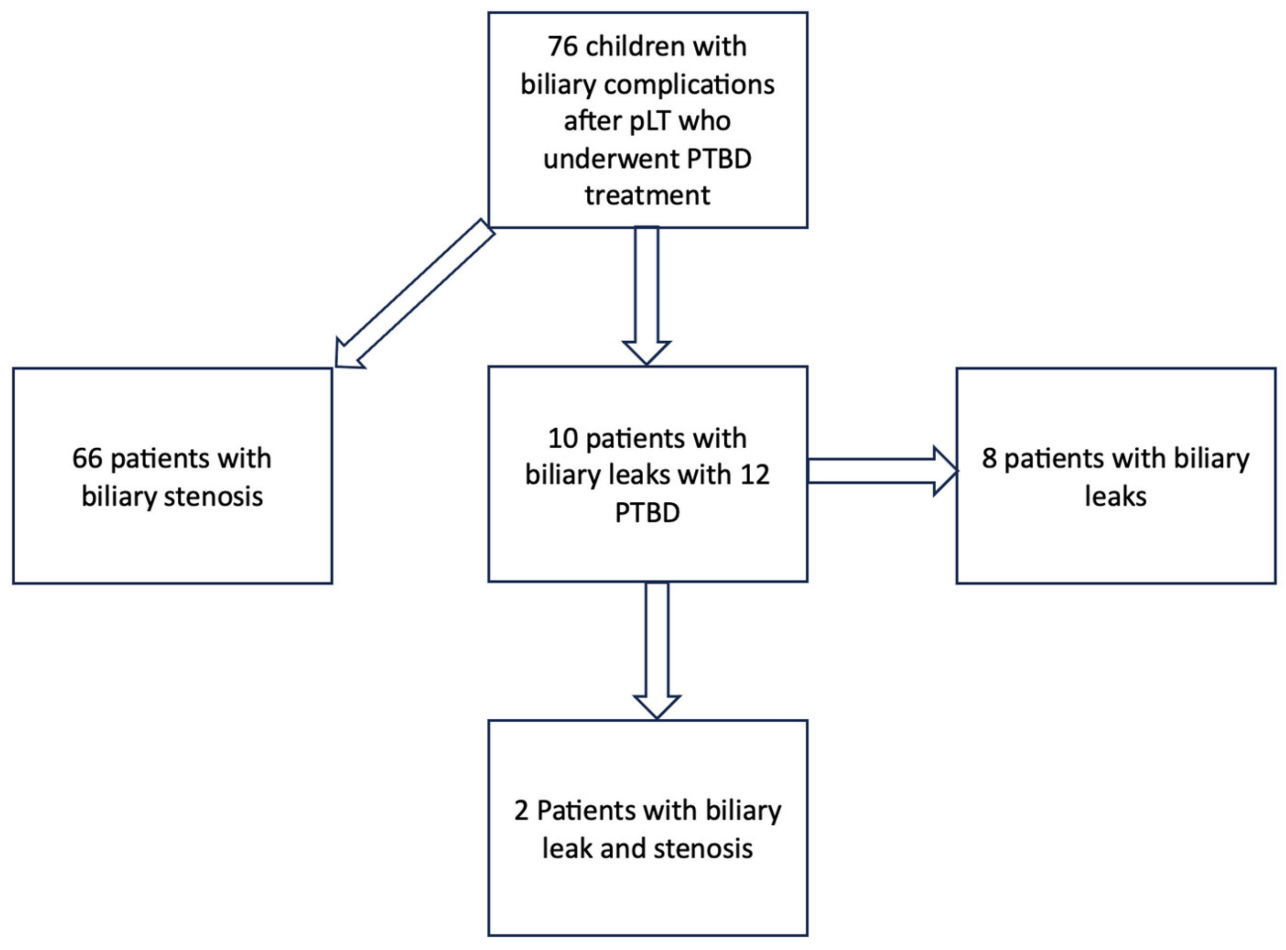
Study cohort of all patients who were treated with PTBD after pLT (pLT: pediatric liver transplantation; PTBD: percutaneous transhepatic biliary drainage).

During the study period, 76 PTBD treatments were performed in pediatric patients after liver transplantation. Indication for PTBD placement was biliary strictures (*n* = 66; 85%) and biliary leakage (*n* = 10; 15%). The study cohort is shown in Scheme 1 and Table 1. Indications for liver transplantation were biliary atresia (39%), metabolic diseases (6%), genetic cholestasis (4%), and miscellaneous (31%). 

### 3.2. Biliary Leaks

**Figure 1:**
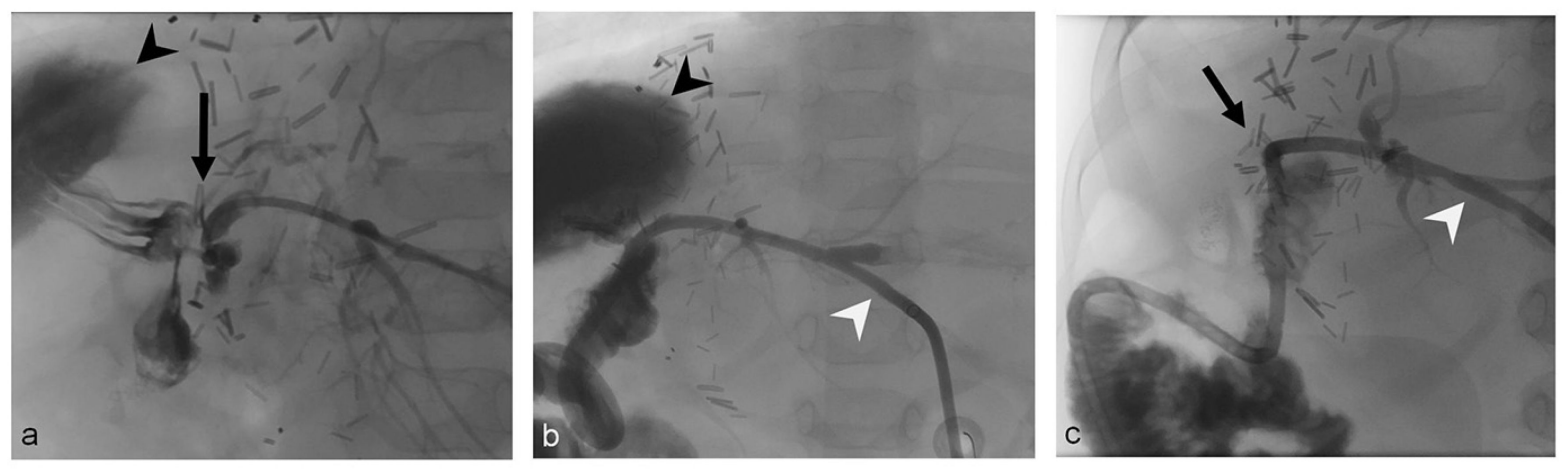
One-year-old boy (Patient 2) with bile leak after liver transplantation (left lateral liver split; deceased donor, end-to-side hepaticojejunostomy). (**a**) The initial cholangiography through the Neff sheath (Cook Medical, Bjaeverskov, Denmark) shows the bile leak (black arrow) and large bilioma (black arrowhead). (**b**) Cholangiography after placement of a 6 F-internal–external PTBD (Cook Medical, Bjaeverskov, Denmark, white arrowhead). (**c**) Cholangiography through an 8 F-Münchner drain (white arrowhead, Peter Pflugbeil GmbH, Zorneding, Germany) 57 days after initial PTBD placement and bilioma drainage detects absent of leak (black arrow) and bilioma.

**Figure 2:**
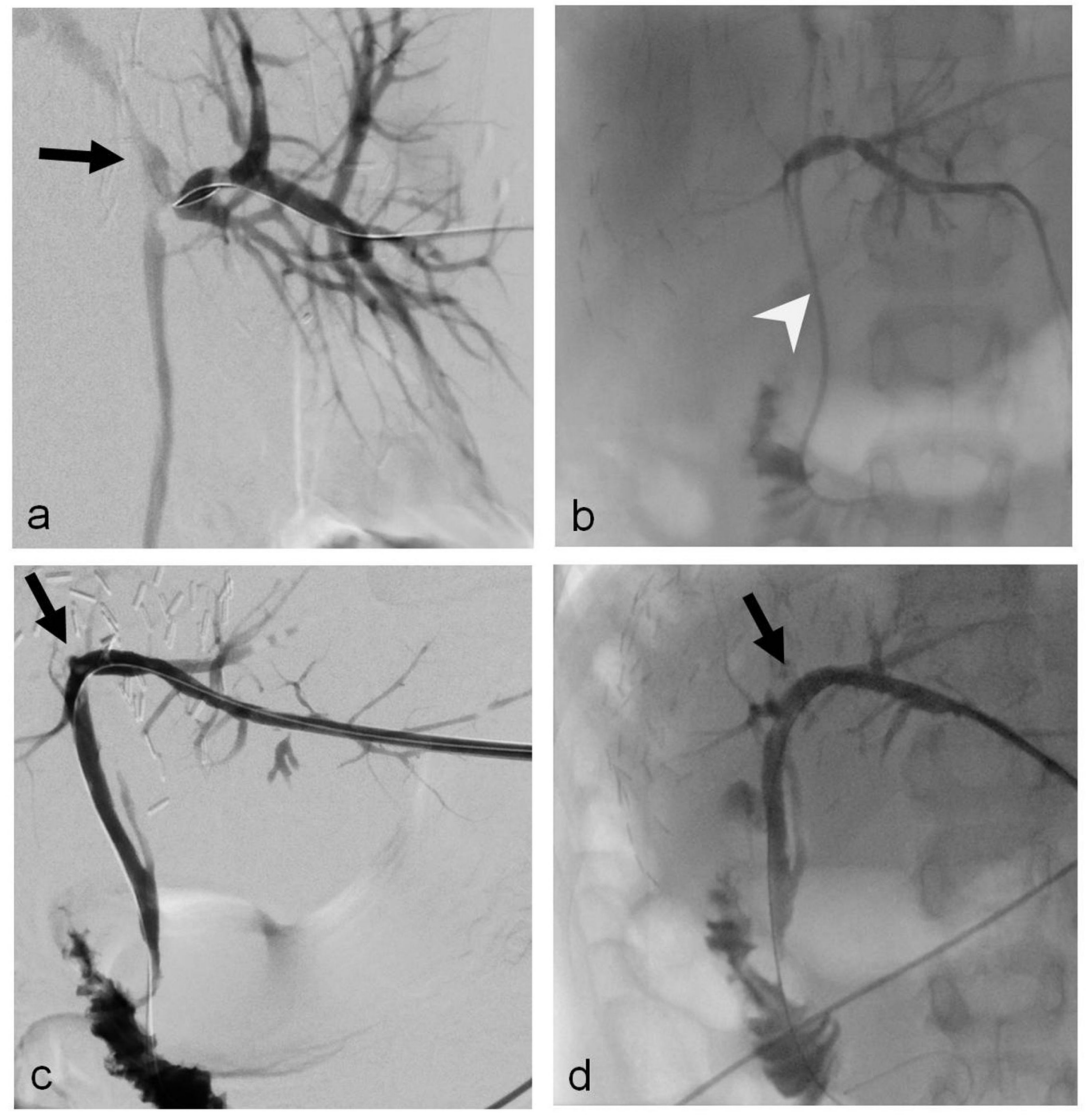
Nine-year-old boy (Patient 4) with a bile leak of the choledocho-choledochostomy after ERCP for biliary stenosis (left liver lobe, living donor). (**a**) The initial cholangiography, detects the bile leak (black arrow) and additional stenosis. (**b**) Cholangiography through the inserted 6 F-internal–external drainage (Cook Medical, Bjaeverskov, Denmark; white arrowhead). (**c**,**d**) Cholangiography through a 10 F-sheath shows resolution of leak and stenosis of the choledocho-choledochostomy (black arrow); bilioma drainage was already removed.

**Figure 3:**
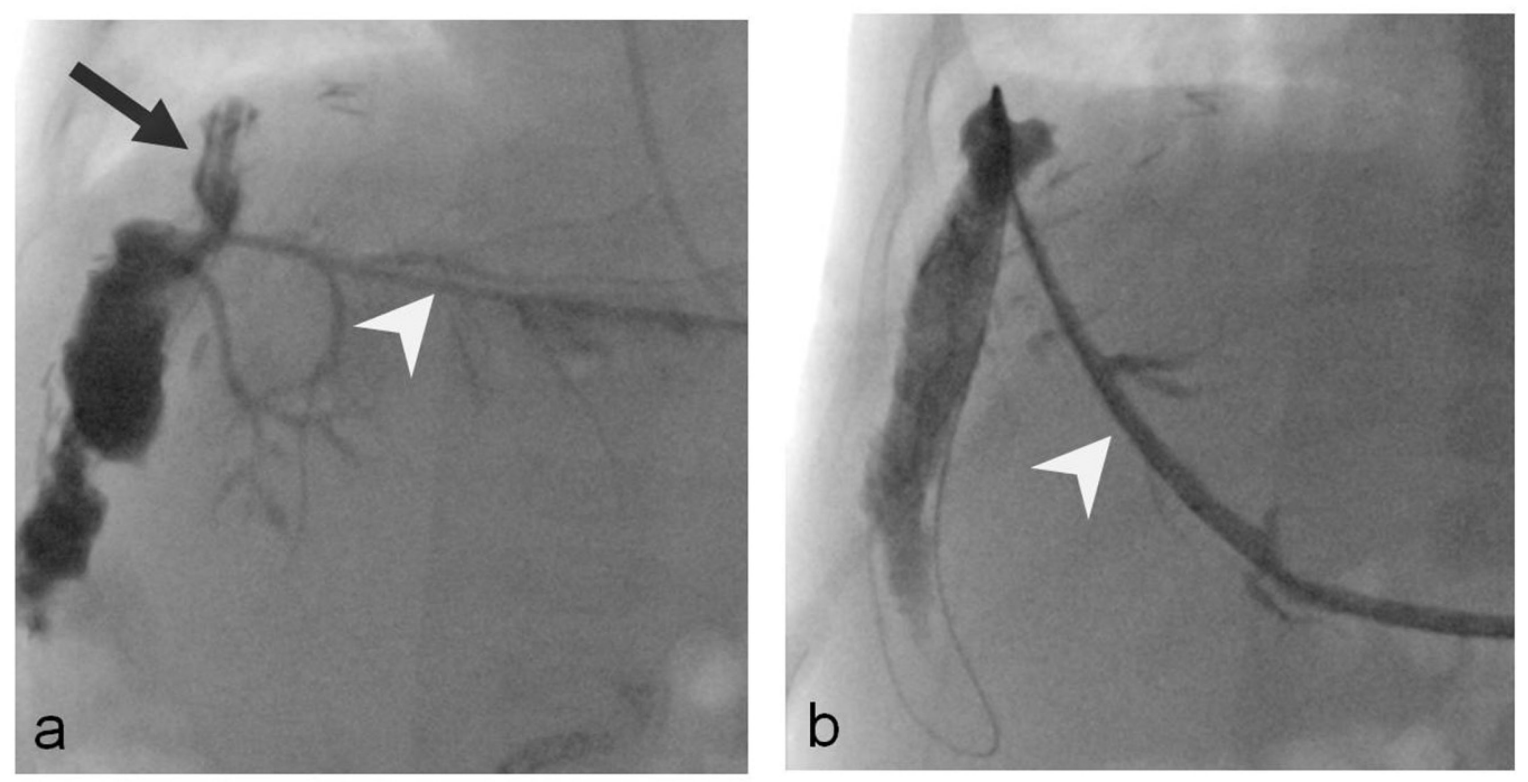
Five-month-old girl (Patient 3) with bile leak at the end-to-side hepaticojejunostomy (left lateral liver split; living donor, double bile duct anastomosis). (**a**) Initial cholangiography, through the Neff sheath (white arrowhead; Cook Medical, Bjaeverskov, Denmark) shows the bile leak located at the hepaticojejunostomy (black arrow). (**b**) Cholangiography through a 10 F-sheath (white arrowhead) 79 days after PTBD placement treatment shows complete resolution of the leak.

The case series included 10 pediatric patients (7 female, 3 male) with PTBD treatment for biliary leakage after liver transplantation. Transplants consisted of a left lateral split (*n* = 6), a left liver lobe (*n* = 2), a right liver lobe (*n* = 1), and a whole liver (*n* = 1). Five transplants were deceased donor transplants and five were living donor transplants. All of these 10 liver transplants were AB0 compatible. The mean weight of the transplants was 508 ± 444.8 g (range: 210–1600 g; median: 347 g). Two patients presented with end-to-end choledocho-choledochostomy (direct duct-to-duct anastomosis), and eight patients presented with end-to-side hepaticojejunostomy with Roux-en-Y limb. A total of 12 PTBDs were placed: one PTD in eight cases and two simultaneous drains in two cases. Three patients presented with simultaneous stenosis of the anastomosis. Bile leak was located at the anastomosis in nine cases. In one case, PTBD was necessary because the central bile duct of segment II had obviously been overlooked during surgery and was not connected to the hepaticojejunostomy. The biliary system was decompressed and not dilated in eight cases. At the start of PTBD treatment, the median age of the patients was 2.5 years (range: 0.5–16.7 years; mean: 6.1 ± 6.4 years), the median height was 82.5 cm (range: 55–172 cm, mean: 105.9 ± 47.3 cm), and the median weight was 10.8 kg (range: 5.1–70.9 kg, mean: 25 ± 23.7 kg). The mean interval between liver transplantation and PTBD treatment for biliary leakage was 40.3 ± 31.7 days (median: 36 days, range: 5–121 days). The overall time of PTBD placement ranged between 15 and 304 days (mean: 109.7 ± 103.6; median: 73). Early biliary complications are often procedure-associated. Accurate preoperative diagnostics can provide surgeons with accurate information about biliary anatomy and thus can help reduce the rate of procedure-related complications and interventions or surgeries required because of the complications. In particular, MRCP has been shown to be an excellent noninvasive method for assessing the anatomy and pathology of the pancreaticobiliary system, relying on the high signal intensity of fluid-containing structures in T2-weighted images [24,25]. The mean time of PTBD treatment of the bile leakage localized at the anastomosis lasted on average for 120.2 ± 104.1 days (median: 89 days; range 20–304 days). The treatment of the leakage due to the unconnected bile duct to the biliary anastomosis lasted for 15 days until surgical revision. The target size of the catheters ranged from 6 to 12 F (mean: 8.6 ± 2; median: 8.5). Additional percutaneous fluid drainage was performed in eight children (80%). Figure 1, Figure 2 and Figure 3 show examples of patients treated with PTBD for biliary leakage. Patient 4 (Figure 2) shows an additional stenosis besides the leakage. Figure 1a, Figure 2a and Figure 3a show the initial cholangiographies through the Chiba needle (Figure 2a) and the Neff sheath (Figure 1a and Figure 1c), respectively. Figure 1b and Figure 2b show the cholangiographies through the internal/external PTBD, and Figure 1c shows it through the Münchner drainage. Figure 2c,d and Figure 3b show the cholangiographies through the 10F sheath with complete healing of the leaks and stenoses. 

### 3.3. Outcome

PTBD placement and treatment of bile leakage at the anastomosis site were technically successful in all patients. In one case, bridging therapy until the surgical connection of the bile duct to the anastomosis was successful through a sufficient external diversion of the bile. No procedure-related complications occurred.

The follow-up period after PTBD removal ranged from 1.7 months to 6.7 years (mean: 3.2 ± 2.2 years; median: 3 years) after the end of PTBD treatment. The patient and transplant survival rate was 100%.

### 3.4. Inflammation Parameters

CRP value decreased significantly after treatment with PTBD. The median CRP before PTBD treatment was 16.6 mg/L and 7.5 mg/L after ending PTBD treatment (*p* = 0.05). Leucocyte counts showed a significant decrease: median of leucocyte counts before PTBD treatment was 10.3 × 10^3^/μL and decreased to 6.4 × 10^3^/μL (*p* = 0.05). The significant decrease in both CRP and leucocyte levels represented strong effects (CRP value: r = 0.53; Leucocyte value: r = 0.53).

### 3.5. Cholestasis Parameters

Total bilirubin showed a significant decrease during therapy with a strong effect size (median before PTBD treatment: 1.3 mg/dL; median after PTBD treatment: 0.5 mg/dL; *p* = 0.006, r = 0.81). Direct bilirubin also decreased significantly during therapy with a strong effect size (*p* = 0.03, r = 0.82). 

The median of gamma-glutamyl transferase GGT before treatment was 104 U/L and decreased significantly to a median of 94.5 U/L after treatment with a strong effect (*p* = 0.009, r = 0.89). Even after the end of therapy, during follow-up, GGT decreased again significantly with a strong effect size (median of last annual follow-up: 26 U/L; *p* = 0.016, r = 0.9). 

The non-statistically significant laboratory value changes can be found in Table 2.

## 4. Discussion

Biliary strictures and biliary leaks are the most common biliary complications after liver transplantation. The mortality rate of biliary complications after pLT is associated with high mortality rates up to 33% and re-transplantation rates between 9 and 16% [18]. A biliary leak is the second most common biliary complication after liver transplantation, with an incidence rate of 2 to 25% after pediatric liver transplantation [18]. In this case series, the median occurrence between liver transplantation and the initiation of PTBD treatment was 36 days. The short period after liver transplantation reflects that the leaks are most likely procedure-associated after surgery.

Percutaneous transhepatic cholangiography (PTC) can demonstrate the biliary leak’s type, location, and severity and allows immediate treatment when combined with PTBD. In this study, leak was already known prior to intervention: all patients showed bile leak during PTC and were treated with consecutive PTBD in the same session. Time of PTBD placement was in mean 40.3 ± 31.7 days (median: 37 days; range: 5–121) after pLT, reflecting the early onset of bile leaks that usually develop within 3 months after pLT [18]. All anastomotic leaks healed with PTBD placement over a mean time of 120.2 ± 104.1 days without requiring surgical revisions.

The course of CRP and leucocyte levels as inflammation parameters demonstrated improvement in inflammation, course of total bilirubin, direct bilirubin, and gamma-glutamyltransferase (GGT), which also proved improvement in liver function during follow-up. In addition to cholangiography and MRCP, statistically significant decreases in inflammation and cholestasis parameters can be used as another diagnostic criterion to prove the occlusion of the bile leak and the success of the treatment with PTBD. Overall, patient and graft survival in our study was 100%.

Hsiao et al. reported a mortality rate of 55.6% (5 of 9 children died) with early biliary complications compared to a mortality rate of 8% (2 of 12 children died) with late complications (more than 3 months after pLT) [18]. The authors included both biliary strictures and leaks [18]. The late complication group did not include biliary leaks, which are a typical early complication after pLT [18]. The early complication group comprised two strictures and seven biliary leaks [18]. Of seven patients with biliary leaks, six died, reflecting this biliary disorder’s severity [18]. Treatment for bile leaks in these seven patients consisted of surgical re-anastomosis (*n* = 1), surgical drainage (*n* = 3), and PTBD in only four cases (57.1%) [18]. Dulcetta et al. recently presented their findings of percutaneous transhepatic cholangiography—a diagnostic tool—in pediatric liver transplants with biliary complications [26]. They found biliary leaks in 6.3% (*n* = 19) of pediatric liver transplants at their center [26]. Many biliary leaks were managed conservatively with indwelling surgical drains (*n* = 4) or the percutaneous drainage of bilioma (*n* = 6) [26]. Interestingly, PTBD was only placed in nine cases of conspicuous or persistent leakage [26]. The outcome of these nine patients remains unclear since the authors documented the overall outcome of all 19 cases: only 11 patients recovered completely without surgery (57%), 5 required surgical revision of biliodigestive anastomosis, and 3 children underwent re-transplantation [26].

The published literature on the interventional radiological treatment of bile leaks after pLT is scarce. Compared to our study, which focused solely on the percutaneous treatments of bile leaks, the existing studies include bile leaks in a more heterogenous study cohort of all possible biliary complications after liver transplantation. Furthermore, these biliary complications were managed differently, with mortality rates higher than presented in this study. Remarkably, some leaks were treated conservatively or with the placement of bilioma drainage solely, despite PTC being performed and detecting biliary leaks. Furthermore, reports did not reveal the location of the bile leak (anastomosis or not). However, concerning the few reports that included PTBD placement for the management of bile leakage after pLT, surgical revision and outcome differed remarkably from our results. Our consequent standard to perform PTC as early as biliary leak is suspected, and to combine the procedure with PTBD placement and additional placement of percutaneous bilioma drainage might be an excellent strategy for a successful treatment strategy and reflects high patient and graft survival in our study. As underlined by the mean duration of 120.2 ± 104.1 days (17.2 ± 14.9 weeks) in our patient cohort, the successful management of anastomotic bile leaks with PTBD requires long therapy. In three of our patients, bile leak was in combination with a stricture, so indwell time was prolonged to treat both leak and finally stricture. Some authors already postulated an overall indwell time of at least 2 months in adult patients after liver transplantation to prevent secondary stenosis. Furthermore, we used rather large draining catheters (up to 12 F) in relation to the body weight of the children to optimize bile diversion and to prevent secondary stricture.

The initial PTBD of non-dilated bile ducts may be difficult, particularly in children, and the risk of hepatic artery injury remains one of the main drawbacks of PTBD performed for non-dilated bile ducts: an incidence of up to 11% has been reported in adult patients [27]. Although we performed PTBD in children with decompressed bile ducts and placed large drainage catheters, we did not experience any complications related to the procedure. To keep the complication rate as low as possible and to reduce the risk of vessel damage, our standard of care was general anesthesia, ultrasound guidance, and puncture of a peripheral bile duct. Puncture of a centrally located bile duct for opacification of the peripheral bile ducts for PTBD placement was never performed.

Darius et al. performed only surgical procedures for treating anastomotic biliary complications [20]. Overall, biliary complications occurred in 98 of 429 pediatric liver transplants (23%) [20]. Of these, 57 were stenoses (57.2%) and 21 were leaks (21.4%; 13 anastomotic and 8 non-anastomotic leakages), and 20 were miscellaneous complications (20.4%) including cholangitis, abscess, hemobilia, secondary biliary cirrhosis, and cholelithiasis. The success rates of surgical repair for biliary anastomotic leakage were 84.6%; 2 of 13 children developed recurrence of leakage [20].

Darius et al. described that 4 of 13 patients required early re-transplantation after surgical treatment of anastomotic bile leaks [20]. A total of 6 of these 13 patients with a bile leak had to undergo a second surgical intervention because of bile fistulas or strictures (*n* = 4) [20]. In addition, the 30-day mortality rate of 3.4% in surgical procedures was higher than the 30-day mortality of 2.5% in PTBD therapy [20,28]. Moreover, serious complications were noted more frequently after surgery than interventional PTBD placement in managing biliary complications after pLT (1.7% vs. 8.5%) [20,28].

Mosconi et al. describe that the clinical success of PTBD treatment for biliary leakage after liver transplantation is only 56%, whereas the clinical success of PTBS treatment for biliary leak after liver resection or cholecystectomy is better (86%) in adult patients. However, the median duration time of PTBS placement was only 21 days [29].

The major limitation of this study is the small number of patients. This is mostly caused by both the relatively small number of liver transplantations in children and the low incidence of bile leaks that do not resolve spontaneously and are in need of interventional treatment.

## 5. Conclusions

The percutaneous transhepatic management of bile leak after pLT is a safe and successful treatment to improve the inflammatory condition, transplant, and patient survival.

## Figures and Tables

**Table 1:** Demographic and clinical data overview of the patients.

Patient	Weight	Sex	Age at the Start of PTBD	Duration of PTBD Treatment	Duration of Follow-Up	Drain for Bilioma	Site of Bile Leak	Target Size of PTBD	Additional Bile Duct Stenosis	Type of Transplant	Living Donor
1	48.9 kg	m	16 y 8 m	7 m (200 d)	63 m (1929 d)	Yes	End-to-side hepaticojejunostomy	10 F	No	Right liver lobe	No
2	9.5 kg	m	1 y 5 m	2 m (57 d)	92 m (2809 d)	Yes	End-to-side hepaticojejunostomy	8 F	Yes	Left lat. split	No
3	5.1 kg	f	5 m	10 m (304 d)	3 m (79 d)	Yes	End-to-side hepaticojejunostomy (Double bile duct anastomosis)	10 F	Yes	Left lat. split	Yes
4	35.9 kg	m	9 y 9 m	8 m (244 d)	24 m (732 d)	Yes	End-to-end choledochcholedochostomy	12 F	Yes	Left liver lobe	Yes
5	6.8 kg	f	8 m	3 m (89 d)	73 m (2216 d)	Yes	End-to-side hepaticojejunostomy (Double bile duct anastomosis)	6 F	No	Left lat. split	No
6	47 kg	f	11 y 7 m	1 m (20 d)	21 m (632 d)	No	End-to-side hepaticojejunostomy	8.5 F	No	Left liver lobe	Yes
7	12 kg	f	3 y 5 m	1 m (25 d)	66 m (1999 d)	Yes	End-to-side hepaticojejunostomy (Double bile duct anastomosis)	10 F	No	Left lat. split	No
8	6 kg	f	6 m	1 m (37 d)	33 m (1002 d)	Yes	End-to-side hepaticojejunostomy (Double bile duct anastomosis)	8.5 F	No	Left lat. split	Yes
9	70.9 kg	f	14 y 8 m	3 m (106 d)	28 m (865 d)	Yes	End-to-end choledochcholedochostomy	10 F	No	Full size	No
10	8.3 kg	f	1 y 6 m	0.5 m (15 d)	60 m (1830 d)	No	Non-anastomosed bile duct of segment II	6 F	No	Left lat. split	Yes

**Table 2:** Laboratory parameters before, directly after, and during follow-up.

Parameter	Before PTBD		After PTBD			Last Annual Follow-Up		
	Median	Mean	Median	Mean	*p*	Median	Mean	*p*
Alkaline Phosphatase (ALP)	519 U/L	494.7 ± 148 U/L	256 U/L	369.1 ± 94.9 U/L	0.5	282 U/L	297.9 ± 47.4 U/L	0.06
Bilirubin Total Direct Indirect	1.3 mg/dL 1.2 mg/dL 0.3 mg/dL	1.7 ± 0.5 mg/dL 1.6 ± 0.5 mg/dL 0.4 ± 0.2 mg/dL	0.5 mg/dL 0.2 mg/dL 0.1 mg/dL	0.46 ± 0.04 mg/dL 0.2 ± 0.03 mg/dL 0.2 ± 0.07 mg/dL	0.006 0.03 0.11	0.53 mg/dL 0.2 mg/dL 0.3 mg/dL	0.5 ± 0.07 mg/dL 0.2 ± 0.02 mg/dL 0.4 ± 0.06 mg/dL	0.12 0.08 0.11
Choline esterase (CHE)	10,638 U/L	10,349 ± 799 U/L	9588 U/L	8744 ± 1118 U/L	0.5	10,376 U/L	10,498 ± 806 U/L	0.12
Gamma-glutamyl transferase (GGT)	104 U/L	177 ± 56.2 U/L	94.5 U/L	121.5 ± 29.3 U/L	0.009	26 U/L	66.8 ± 21.6 U/L	0.016
Glutamate dehydrogenase (GLDH)	4.1 U/L	7 ± 2.3 U/L	4.3 U/L	4.9 ± 0.92 U/L	0.23	4.0 U/L	27.8 ± 23.8 U/L	0.917
Aspartate transaminase (AST)	20 U/L	48.9 ± 19.1 U/L	32.5 U/L	42.9 ± 9 U/L	0.15	31 U/L	54.9 ± 25.8 U/L	0.43
Alanine transaminase (ALT)	42 U/L	79.7 ± 37.8 U/L	33.5 U/L	45.2 ± 12.8 U/L	0.25	30.5 U/L	40.2 ± 9.2 U/L	0.42
Albumin	24.1 g/L	24.3 ± 2.3 g/L	31.7 g/L	31.8 ± 3.3 g/L	0.14	36.6 g/L	36.8 ± 1.2 g/L	0.29
INR	1.1	1.1 ± 0.04	1.1	1.1 ± 0.02	0.36	1.0	0.9 ± 0.02	0.11
Quick	88%	87.7 ± 5.3%	86%	84.8 ± 3.8%	0.36	101%	99.6 ± 2.8%	0.11
CRP	16.6 mg/L	47.2 ± 16.4 mg/L	7.5 mg/L	14.3 ± 4.8 mg/L	0.05	2.8 mg/L	4.8 ± 1.8 mg/L	0.11
Leucocytes	10.3 × 10^3^/μL	9.6 ± 1.3 × 10^3^/μL	6.4 × 10^3^/μL	6.7 ± 0.8 × 10^3^/μL	0.05	6.6 × 10^3^/μL	7.4 ± 1.1 × 10^3^/μL	0.14

## Data Availability

The data underlying the case series are stored in an internal database.
